# The COVID-19 pandemic influenced the temporal dynamics of antimicrobial resistance markers and bacterial community across urban wastewater treatment plants

**DOI:** 10.1007/s42770-026-02069-6

**Published:** 2026-08-31

**Authors:** Celso G. B. Filho, Francisca A. S. Oliveira, Victor L. M. Rodrigues, Denise C. Hissa, Maisa V. H. Barros, Allysson A. de Farias, Gabriel T. Dantas, André B. dos Santos, Julio C. M. Ximenes, Vânia M. M. Melo

**Affiliations:** 1 Department of Civil Construction, Federal Institute of Education, Science and Technology of Ceará, Morada Nova, Ceará, Brazil; 2 Doctoral Program in Biotechnology of the Northeast Biotechnology Network (RENORBIO), Federal University of Ceará, Fortaleza, Ceará Brazil; 3 Department of Biology, Federal University of Ceará, Fortaleza, Ceará Brazil; 4 Center for Genomics and Bioinformatics (CeGenBio), Center for Research and Drug Development, Federal University of Ceará, Fortaleza, Ceará Brazil; 5 Christus University Center (Unichristus), Fortaleza, Ceará Brazil; 6 Department of Hydraulic and Environmental Engineering, Federal University of Ceará, Fortaleza, Ceará Brazil; 7 Pasteur Fiocruz Center for Immunology and Immunotherapy, Fiocruz- Ceará, Eusébio, Ceará Brazil

**Keywords:** Wastewater surveillance, Antimicrobial resistance markers, Quantitative PCR, 16S rRNA metabarcoding, Wastewater treatment plants

## Abstract

Urban wastewater systems represent important interfaces between human activity and the environmental occurrence of antimicrobial resistance (AMR) markers. We assessed the temporal dynamics of *intI1*, *ermB*, and the 16 S rRNA gene by quantitative PCR across three wastewater systems (EPC, CJC, and JW) in Fortaleza, Brazil, from November 2021 to November 2023. Bacterial communities were additionally characterized by 16 S rRNA gene metabarcoding in 18 samples collected in December 2021 and January 2022. A synchronized decline in 16 S rRNA gene and *intI1* concentrations beginning in late 2022 was observed across all three wastewater systems, suggesting a shift toward lower microbial abundance. The *ermB* gene showed higher and more variable concentrations during part of the pandemic period, followed by convergence toward lower levels; however, the absence of antimicrobial-consumption data precluded attribution of this pattern to changes in macrolide selective pressure. Normalized antimicrobial resistance marker abundances were comparatively stable at EPC and JW but more variable at CJC. EPC exhibited the highest ASV richness, whereas CJC and JW showed greater diversity according to Shannon and inverse Simpson indices. Beta-diversity analyses identified wastewater system as the principal factor associated with bacterial community structure, while the effect of sampling period was smaller and metric-dependent. Neither *ermB* nor *intI1* was individually associated with community composition, although *intI1* showed a limited effect after adjustment for wastewater system in one model. Physicochemical parameters were not significantly associated with normalized marker abundances in the exploratory paired analysis. *Arcobacter*, *Acinetobacter*, and other potentially relevant genera were detected, but no direct associations between these taxa and the monitored AMR markers could be established. These findings highlight the value of integrating longitudinal qPCR, microbiome profiling, and environmental characterization to improve the interpretation of targeted AMR markers in One Health wastewater surveillance.

## Introduction

Urban wastewater is a highly heterogeneous matrix that aggregates microbial consortia from households, hospitals, industrial discharges, and urban runoff [1]. Within this mixture, antibiotic-resistant bacteria (ARB) and antibiotic resistance genes (ARGs) are increasingly prevalent, largely driven by expanding human activities and antimicrobial consumption [2, 3]. Wastewater also concentrates emerging contaminants, including antimicrobials, detergents, disinfectants, biocides, industrial chemicals, and trace metals, that can reshape microbial composition and physiology [4, 5]. Continuous exposure to these stressors imposes selective pressures that favor the persistence and dissemination of microorganisms and resistance determinants [6, 7]. As a result, sewage systems operate as dynamic reservoirs where resistance evolution and horizontal gene transfer may be intensified, reinforcing their value for antimicrobial resistance (AMR) surveillance [8].

Antibiotic resistance is a major global public health threat, undermining medical advances, food production systems, and sustainable development [3, 9–11]. Its emergence reflects prolonged selective exposure to antimicrobials and the accumulation of genetic and metabolic mechanisms that erode therapeutic efficacy [12]. Because ARB and ARGs circulate across human, animal, and environmental compartments, their occurrence in domestic sewage highlights the interconnected dynamics central to One Health [2, 13–15]. Accordingly, wastewater surveillance has expanded across Europe, Asia, and North America as a strategic approach to monitor resistance circulation and evolution [16–20]. In Brazil, however, data on ARB/ARG burdens in untreated and treated sewage, and in receiving waters, remain limited, particularly in regions with inadequate sanitation infrastructure where environmental dissemination may be amplified [21–23].

The COVID-19 pandemic represented an unprecedented global disruption that altered antimicrobial consumption patterns and influenced the environmental dynamics of antimicrobial resistance [24]. During the early and peak phases, misinformation and precautionary clinical practices contributed to increased prescription and misuse of antibiotics despite the viral etiology, while hospital admissions and intensified disinfectants use further amplified selective pressures in urban wastewater [25–29]. These concurrent changes make the pandemic a large-scale anthropogenic disturbance through which abrupt shifts in human behavior, clinical practice, and chemical inputs can be examined in relation to dynamics of resistance.

Because these inputs converge in sewer networks, wastewater treatment plants (WWTPs) serve as strategic interfaces between human activity and receiving ecosystems and have been widely adopted for wastewater-based epidemiology [30]. Although microbial communities and ARG profiles have been extensively surveyed, most studies remain cross-sectional and provide limited insight into long-term trajectories, particularly in tropical urban settings and during major disruptions such as the pandemic [29]. In Brazil, where sanitation coverage remains insufficient and only ~ 45% of generated sewage is treated across approximately 2800 operational WWTPs [31, 32], infrastructural heterogeneity and year-round climatic conditions may shape resistance persistence in ways that differ from temperate regions [33].

Considering the far-reaching ecological, clinical, and economic consequences of antimicrobial resistance, and guided by the One Health framework that emphasizes integrated environmental surveillance, monitoring AMR markers, class 1 integrons, and bacterial community composition in urban wastewater has become increasingly important [20, 34]. However, it remains unclear how WWTPs influence the persistence and temporal dynamics of these molecular markers during periods of intensified antimicrobial consumption, such as the COVID-19 pandemic, and whether they act primarily as reservoirs or ecological filters within urban microbial ecosystems. Addressing these questions requires longitudinal investigations that integrate bacterial community composition with quantitative analyses of targeted AMR markers under real-world operational conditions.

To address these knowledge gaps, this study investigated the occurrence and temporal dynamics of targeted antimicrobial resistance markers and associated bacterial communities in full-scale WWTPs in the municipality of Fortaleza, Ceará State, Brazil, during and after the COVID-19 pandemic. Quantitative PCR (qPCR) was used to quantify the abundance of the class 1 integron integrase gene (*intI1*), the erythromycin resistance gene (*ermB*), and the 16 S rRNA gene, whereas 16 S rRNA gene amplicon sequencing was used to characterize bacterial community composition, assess microbial diversity, and identify genera containing opportunistic pathogens. We hypothesized that the COVID-19 pandemic, as a large-scale societal disturbance, influenced the temporal dynamics of the targeted antimicrobial resistance markers *ermB* and *intI1* and bacterial community composition in urban wastewater treatment plants. We further hypothesized that these temporal responses would persist beyond the acute phase of the pandemic and vary among wastewater treatment plants. By integrating molecular surveillance with ecological analyses across a pandemic-driven temporal gradient, this study provides new insights into how large-scale societal disruptions influence the dynamics of the evaluated molecular markers and microbial community structure in tropical urban wastewater systems. These findings are particularly relevant for strengthening national sanitation strategies and advancing One Health–based AMR surveillance frameworks in Brazil.

## Materials and methods

### Wastewater sampling and preparation of filtration membranes

For the surveillance of AMR markers, we employed the wastewater monitoring infrastructure previously established during the COVID-19 pandemic for SARS-CoV-2 detection. Wastewater samples were collected by the Ceará State Water and Sewage Company (CAGECE) from November 2021 to November 2023 using two types of automated samplers. The Etsus 10,000 was used at sites with hydraulic heads up to 4.0 m, whereas the ISCO 3700 was deployed at locations requiring greater pumping capacity, reaching hydraulic heads up to 8.0 m. To obtain representative composite samples, the samplers were programmed to collect grab samples every 20 min and pool them into a single container kept at 4 °C. At the end of each sampling period, the composite sample was homogenized and transferred to refrigerated storage until laboratory processing.

Aliquots of 100 mL were transferred to bottles containing 1 mL of MgCl₂ (2.5 M). Samples were then acidified with acetic acid (1 M) to a pH of 3.0–3.5 and filtered through sterile cellulose ester membranes (0.45 μm pore size, 47 mm diameter; Merck Millipore Ltd.). The filtered volume was recorded to allow subsequent calculation of gene copy numbers by qPCR. Membranes were stored at − 80 °C until DNA extraction.

### Selection of samples

Wastewater samples were collected from the Pre-Conditioning Station (EPC) and from the Conjunto Ceará (CJC) and José Walter (JW) wastewater treatment plants (WWTPs). Filtration membranes corresponding to the period from November 2021 to November 2023 were selected, with samples strategically distributed to represent the pandemic phase of COVID-19 (before May 2022, the official end of the pandemic in Brazil) and the post-pandemic phase (after May 2022).

### DNA extraction for qPCR and 16 S rRNA gene sequencing

For processing membranes for qPCR analysis, sterile microtubes were prepared with ~ 0.5 mL of 1 mm zirconia beads. Using sterile forceps, each filtration membrane was transferred from the original collection tube into the prepared microtube. Subsequently, 600 µL of MW1 reagent (DNA/RNA MGIEasy Magnetic Beads) and 6 µL of β-mercaptoethanol were added. Mechanical disruption was performed using a BeadBeater homogenizer (Biospec Products, USA) with four cycles of 30 s agitation, interspersed with cooling on ice to prevent nucleic acid degradation (total lysis time: 2 min). After homogenization, samples were centrifuged at 18,000 × *g* for 15 min at 20 °C, and the supernatant was transferred to clean microtubes for nucleic acid extraction. DNA extraction was carried out using the automated MGISP-960 system (MGI Tech) with the MGIEasy Magnetic Beads DNA/RNA extraction kit, following the manufacturer’s instructions. DNA concentration and purity were assessed using a NanoDrop^®^ ND-1000 spectrophotometer (Thermo Fisher Scientific, Wilmington, DE, USA) by measuring absorbance at 260 and 280 nm.

Meanwhile, DNA extraction for 16 S rRNA gene sequencing was conducted using the AllPrep PowerViral DNA/RNA kit (Qiagen^®^, Germany) with 2-mercaptoethanol (Sigma-Aldrich^®^, Germany), in accordance with the manufacturer’s protocol, with minor modifications. The fragmentation of the membrane was realized using a BeadBeather (Biospec Products, USA) with five two-minute cycles. During this process, the samples were kept on ice. Following the fragmentation process, the samples were centrifuged at 18,000 x *g* for 15 min at 20 °C. The samples were eluted in 100 µL of nuclease-free water and quantified using the Nanodrop 1000 (Thermo Fisher Scientific).

### Quantification and statistical analysis of AMR markers

Quantitative polymerase chain reaction PCR (qPCR) was used to quantify the absolute abundance of *intI1*, *ermB* and 16 S rRNA, with results expressed as log₁₀ copies mL⁻¹.

The longitudinal qPCR dataset comprised 14 sampling points for each WWTP, including five points assigned to the pandemic phase and nine assigned to the post-pandemic phase, according to the temporal classification adopted in this study. The same sampling points were evaluated at EPC, CJC, and JW, resulting in 42 environmental samples. Sampling was not performed at fixed monthly or biweekly intervals. Instead, dates were selected from the available wastewater surveillance archive to represent epidemiologically relevant periods, with particular attention to periods of elevated COVID-19 incidence. Each environmental sample was analyzed in technical triplicate by qPCR; these technical replicates were used to assess analytical variability and were not considered independent temporal observations.

The molecular targets were selected according to their relevance to the study objectives and to antimicrobial consumption patterns reported in Brazil and worldwide [9]. The *ermB* gene was included as a marker of macrolide resistance because of the extensive use of this antimicrobial class, particularly during the COVID-19 pandemic, whereas *intI1* was selected as a marker of class 1 integrons and anthropogenic influence in wastewater environments. Because this targeted qPCR approach included only one ARG and one integron-associated marker, it was not intended to characterize the complete wastewater resistome. Accordingly, the results were interpreted as marker-specific temporal dynamics rather than as comprehensive resistome profiles.

In addition to absolute quantification, the abundances of *ermB* and *intI1* were normalized to the 16 S rRNA gene and expressed as copies per cell equivalent, following the approach described by Chen et al. [35].

qPCR assays were performed on a QuantStudio™ 3 Real-Time PCR System (Thermo Fisher Scientific, USA) using 96-well plates with a final reaction volume of 20 µL, consisting of 10 µL of iTaq Universal SYBR Green Supermix (Bio-Rad), 5 µL of DNA template, and variable volumes of the forward and reverse primers to obtain final concentrations ranging from 250 to 400 nM. DNase-free deionized water was added to complete the reaction volume. All reactions were run in technical triplicate, with positive and negative controls included. Primer sequences and qPCR amplification conditions were adopted from Leroy et al. (2022) and are summarized in Table 1 [36]. Standard curves for absolute quantification were constructed using gBlocks for each target gene, with ten-fold serial dilutions prepared to generate the calibration curves. All assays displayed strong linearity, with correlation coefficients (R²) > 0.99, slopes ranging from − 3.32 to − 3.40, and amplification efficiencies between 96.6% and 99.99%.

**Table 1:** Primer sequences and qPCR conditions used in the study

Gene	Primers	Amplicon (bp)	Conditions	Primer Concentration (nM)	Efficiency (%)	*R*^2^
16 S rRNA	1055-F ATGGCTGTCGTCAGCT 1392-R ACGGGCGGTGTGTAC	337	95 °C for 10 min (1 cycle); 95 °C for 15 s, 50 °C for 1 min and 72 °C for 1 min (30 cycles)	250	99.99	0.99
*intI1*	*intI1*-F CCTCCCGCACGATGATC *intI1*-R TCCACGCATCGTCAGGC	280	95 °C for 10 min (1 cycle); 95 °C for 15 s, 55 °C for 30 s and 72 °C for 10 s (40 cycles)	400	96.76	0.99
*ermB*	*ermB*-F CGTGCGTCTGACATCTATCTGA *ermB*-R CTGTGGTATGGCGGGTAAGTT	190	94 °C for 5 min (1 cycle); 94 °C for 30 s, 55 °C for 1 min and 72 °C for 30 s (40 cycles)	300	97.08	1

*F* forward primer, *R* reverse primer, *bp* base pairs

For qPCR data, graphs were generated using OriginPro 2025 (OriginLab Corporation, 2025). Statistical analyses were performed in R version 4.3.2 (R Core Team, 2024) using the *tidyverse*, *rstatix*, *ggplot2*, *lubridate*, and *stats* packages. Data normality and homogeneity of variances were assessed using the Shapiro–Wilk and Levene’s tests, respectively. Because the parametric assumptions were met, temporal comparisons were performed separately for each gene and WWTP using one-way ANOVA followed by Tukey’s post hoc test. Statistical significance was established at Tukey-adjusted *p* < 0.05.

### Physicochemical characterization and exploratory association with AMR markers

Physicochemical characterization of wastewater samples was performed according to the *Standard Methods for the Examination of Water and Wastewater* (APHA, 2012) [37]. The evaluated parameters were pH, determined according to method 4500-H⁺ B; ammonia, determined according to method 4500-NH₃ B; and chemical oxygen demand (COD), determined according to method 5220 B.

The relationships between the physicochemical parameters and the normalized abundances of *ermB* and *intI1* were evaluated using samples for which paired qPCR and physicochemical measurements were available. This analysis included six paired observations obtained in December 2021 and January 2022. Marker abundances were normalized to the 16 S rRNA gene using the difference between the log₁₀-transformed values, as described in the preceding section:

$$\:AR{G}_{norm}={\mathrm{l}\mathrm{o}\mathrm{g}}_{10}\left(ARG\right)-{\mathrm{l}\mathrm{o}\mathrm{g}}_{10}\left(16S\right)$$

Associations between pH, COD, and ammonia and the normalized abundance of each marker were initially assessed using Spearman’s rank correlation coefficient (ρ). Separate simple linear regression models were subsequently fitted using each physicochemical parameter as the explanatory variable and the normalized abundance of *ermB* or *intI1* as the response variable. All analyses were conducted in R version 4.5.2. Statistical significance was established at *p* < 0.05. Because only six paired observations were available, the correlation and regression analyses were considered exploratory, and their effect estimates were interpreted together with the corresponding coefficients and measures of explained variance rather than solely according to statistical significance.

### 16S rRNA gene library preparation and sequencing

Taxonomic characterization of the bacterial community was performed through *16 rRNA* gene metabarcoding sequencing on the Illumina MiSeq platform. A total of 18 samples were analyzed, comprising three experimental groups corresponding to the EPC, CJC, and JW wastewater treatment systems, with six samples per group. Sampling was conducted in December 2021 and January 2022, with three biological replicates collected per group during each sampling period.

Amplicon libraries were generated in technical triplicate for each sample, targeting the V4 region of the *16 rRNA* gene amplified by PCR using primers 515 F-Y and 806-R [38, 39]. PCR reactions were carried out in 30 µL containing 2 µL of genomic DNA (5 ng/µL), 0.75 µL of each primer (10 µM), 15 µL of KAPA HiFi 2× ReadyMix (Roche), and 11.5 µL of nuclease-free water. Amplifications were performed on a Veriti 96-well Thermal Cycler (Applied Biosystems) under the following conditions: initial denaturation at 95 °C for 3 min, followed by 35 cycles of 98 °C for 20 s, 55 °C for 30 s, and 72 °C for 30 s, with a final extension at 72 °C for 5 min. Amplicons were verified on 2% agarose gels and purified using AMPure XP beads (Beckman Coulter). A second PCR was performed for library indexing, using 25 µL of KAPA HiFi 2× ReadyMix, 5 µL of each Nextera XT index (Illumina), 5 µL of purified amplicon, and 10 µL of water. Indexed libraries were purified again using AMPure XP beads. Libraries were quantified fluorometrically with a Qubit 2.0 fluorometer using the dsDNA BR Assay Kit (Thermo Fisher Scientific), and fragment size distribution was assessed on a TapeStation 4150 (Agilent) using the DNA HS D1000 kit. Libraries were then diluted and pooled in equimolar concentrations, adjusted to 10 pM, and denatured with 20% PhiX (Illumina).

### Bioinformatic processing and taxonomic profiling

Raw 16 S rRNA gene amplicon sequences were first trimmed using fastp (v0.23.4) [35] to remove primers and low-quality bases, applying a minimum Phred quality threshold of 20, a minimum read length of 100 bp, and excluding reads containing ambiguous bases. The resulting reads were processed using the DADA2 pipeline (v1.16) [40], including quality filtering, error modeling, denoising, paired-end read merging, chimera removal, and amplicon sequence variant (ASV) inference. Taxonomic assignment of ASVs was performed using a naïve Bayesian classifier trained on the corresponding primer region against the SILVA database (v138.2) [41]. No minimum sequencing-depth threshold was established for sample inclusion.

Relative abundances of bacterial taxa were calculated from the ASV feature table using the phyloseq package (v1.56.0) in R environment. Taxonomic composition profiles were visualized using stacked bar plots generated with the ggplot2 package (v4.5.3), showing the relative abundance of bacterial phyla across treatments. Phyla with relative abundances below 0.5% were grouped into the category “Rare” for visualization purposes. A heatmap of bacterial genera was generated using taxa with relative abundance > 0.01. Sample clustering was performed based on Weighted UniFrac distances using hierarchical clustering with the UPGMA (average linkage) method, whereas bacterial genera were clustered using Euclidean distance with complete linkage. Sample annotations indicating wastewater treatment plant (WWTP) and sampling period were included above the heatmap. The heatmap was generated using the ComplexHeatmap package (v2.18.0), supported by the circlize package (v0.4.16), in the R environment (v4.5.3).

The relative abundance of bacterial genera was compared between the D21 and J22 sampling periods across the different WWTPs (EPC, CJC, and JW). Data were grouped by genus, WWTP, and sampling date, and mean relative abundances were calculated for each group. The percentage change between periods was calculated as:

$$\:\mathrm{Variation\:(\%)}=\frac{J22-D21}{D21}\times\:100$$

Positive values indicated an increase, whereas negative values indicated a reduction in relative abundance between sampling periods.

### Statistical analyses of qPCR and microbial community data

Alpha diversity of bacterial communities was assessed based on richness and diversity metrics calculated from ASV abundance matrices. Observed richness (Observed ASVs), estimated richness (Chao1), Shannon diversity index, and inverse Simpson diversity index (InvSimpson) were calculated to evaluate differences among WWTPs and sampling periods. ACE estimated richness, Simpson diversity, and Fisher’s alpha were additionally calculated to provide descriptive measures of alpha diversity across the complete dataset. These pooled metrics were not used for statistical comparisons among WWTP–period groups. Differences among groups were assessed using one-way ANOVA for normally distributed data or Kruskal–Wallis tests for non-normally distributed data. For significant ANOVA results, pairwise comparisons were performed using Student’s t-test, whereas significant Kruskal–Wallis results were followed by Dunn’s post hoc test. P-values from pairwise comparisons were adjusted using the Benjamini–Hochberg (BH) correction for multiple testing. Alpha diversity analyses and statistical procedures were conducted in the R environment using the phyloseq (v1.52.0), vegan (v2.6–8), car (v3.1–3), and ggpubr (v0.6.0) packages. Statistical significance was considered at *p* < 0.05.

Microbial community structure was assessed through beta diversity analyses using Bray–Curtis, Unweighted UniFrac, and Weighted UniFrac distance metrics. Distance matrices were evaluated using permutational multivariate analysis of variance (PERMANOVA), with WWTP and sampling date included as explanatory variables. Marginal tests were performed to assess the individual effects of each factor, and the proportion of explained variation was estimated using the coefficient of determination (R²), based on 999 permutations. The potential influence of differences in multivariate dispersion among groups was evaluated using PERMDISP through both parametric and permutation-based approaches. Community separation was further assessed using ANOSIM with 999 permutations. Principal Coordinates Analysis (PCoA) was performed using the three distance matrices, and ordination plots were generated with 95% confidence intervals according to WWTP grouping. The Weighted UniFrac-based PCoA was selected for graphical visualization. Beta-diversity analyses were conducted in R version 4.5.2 using phyloseq (v1.52.0) and vegan (v2.6–8). Statistical significance was established at α = 0.05.

### Association between bacterial community composition and AMR markers

The association between bacterial community composition and the abundances of *ermB* and *intI1* was evaluated using Bray–Curtis distance matrices. This analysis included only samples with paired microbiome and qPCR data, comprising 15 samples from EPC, CJC, and JW. The JW-D21 sampling event was excluded because corresponding qPCR measurements were unavailable, reducing the dataset from 18 to 15 samples for this analysis.

The abundances of *ermB* and *intI1* were log₁₀-transformed before their inclusion as explanatory variables in PERMANOVA models. The effects of WWTP and sampling period were first evaluated in the paired dataset using marginal tests. Each marker was then evaluated individually to determine its unadjusted association with bacterial community composition. Additional marginal models were fitted to estimate the contribution of each marker after accounting for WWTP. Finally, a combined model including WWTP, *ermB*, and *intI1* was used to evaluate the independent contribution of each marker when both were included simultaneously. The proportion of variation explained by each variable was estimated using R², and statistical significance was assessed using 999 permutations. Analyses were conducted in R version 4.5.2 using phyloseq (v1.52.0) and vegan (v2.6–8). Statistical significance was established at *p* < 0.05.

## Results and discussion

### Temporal dynamics of resistance markers

**Fig. 1:**
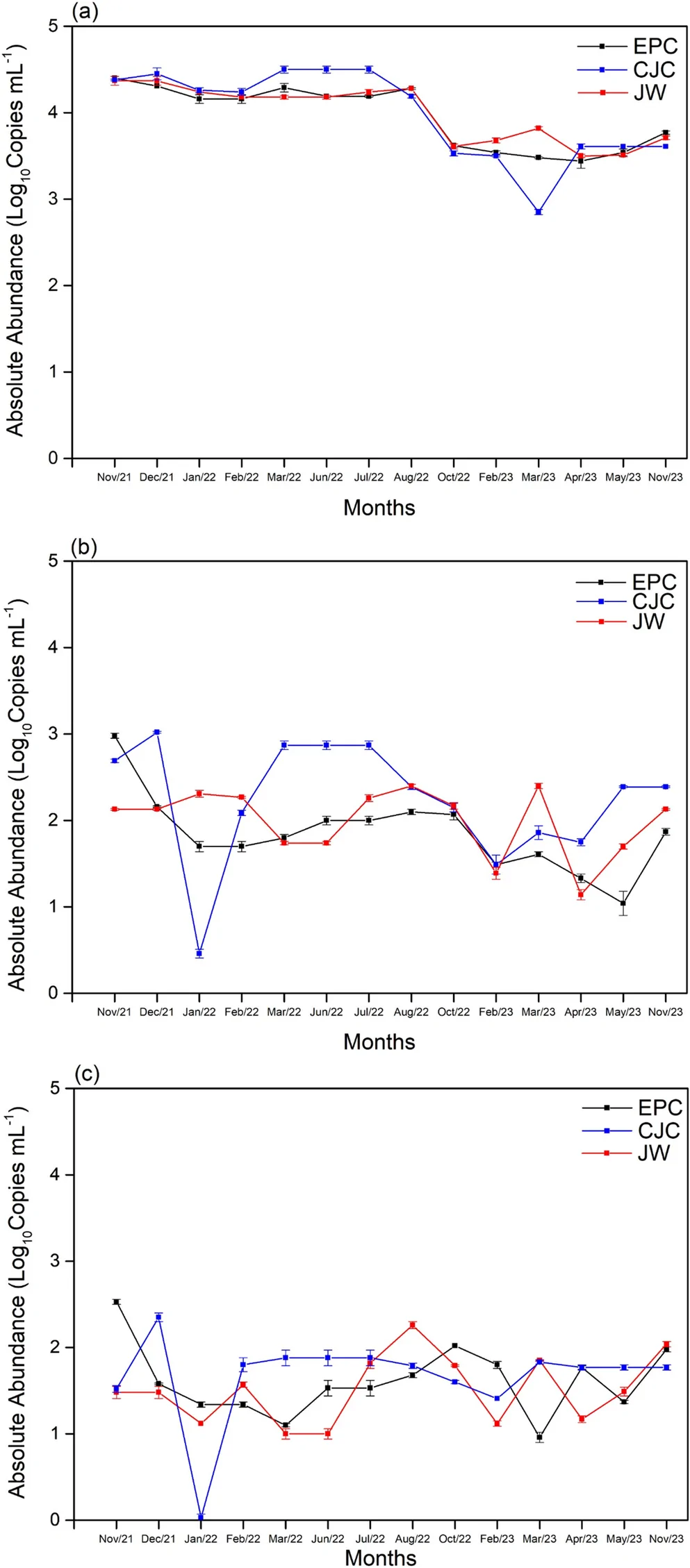
Absolute abundance (log₁₀ copies mL⁻¹) of the 16 S rRNA gene (**a**), *intI1* (**b**), and *ermB* (**c**) quantified by qPCR in wastewater samples collected from the EPC, CJC, and JW treatment plants between November 2021 and November 2023. Data points represent the mean of technical replicates and error bars indicate standard deviation

All three wastewater treatment plants (WWTPs) exhibited a comparable initial regime for the *16 rRNA* gene between November 2021 and March 2022 (*p* < 0,001 for all WWTPs), with concentrations ranging from 4.2 to 4.5 log copies mL⁻¹. During this early interval, only minor inter-plant deviations were observed, with CJC reaching the highest value (~ 4.5 log copies mL⁻¹ in February 2022) while EPC and JW remained between 4.3 and 4.4 log copies mL⁻¹ (Fig. 1a). The narrow dispersion across plants suggests broadly similar bacterial biomass loads entering the systems during this period, consistent with previous observations that *16 rRNA* represents a robust proxy for total microbial abundance in wastewater matrices [36, 42].

A marked transition occurred between August and October 2022, when 16 S rRNA gene concentrations declined within all three WWTPs (all adjusted *p* < 0.001) and converged to approximately 3.5–3.7 log copies mL⁻¹. Compared with August 2022, 16 S rRNA gene concentrations remained lower at all sampling points from February to November 2023 in the three WWTPs (all adjusted *p* < 0.001), fluctuating between 2.8 and 3.6 log copies mL⁻¹ (Fig. 1a). Temporal variability of this magnitude is consistent with longitudinal wastewater studies demonstrating that microbial markers can undergo regime shifts driven by large-scale changes in influent composition and hydraulic dilution [43]. This system-wide transition coincides temporally with the post-pandemic phase, following the period of extensive macrolide consumption associated with COVID-19 management [24]. Although causality cannot be directly established, the synchronization across plants suggests that large-scale shifts in population-level antibiotic exposure may have contributed to the observed regime change [44, 45].

The temporal trajectory of the *intI1* gene showed some visual parallels with that of the *16 rRNA* gene but included more pronounced plant-specific fluctuations. Relatively high *intI1* abundances were observed at selected sampling points between November 2021 and March 2022. Between August and October 2022, concentrations declined at CJC (adjusted *p* = 0.0031) and JW (adjusted *p* < 0.001), whereas no difference was detected at EPC (adjusted *p* > 0.999). Lower concentrations were subsequently observed at plant-specific sampling points in early 2023, followed by distinct temporal patterns among WWTPs. At CJC, *intI1* concentrations showed a pronounced transient decrease in January 2022 (~ 0.45 log copies mL⁻¹), with values lower than those recorded in December 2021 and February 2022 (both adjusted *p* < 0.001). (Fig. 1b), coinciding with a simultaneous drop in *ermB* (all adjusted *p* < 0.001) (Fig. 1c). Similar abrupt excursions have been documented in full-scale systems and are frequently attributed to transient shifts in influent composition or episodic dilution events [43, 46]. Importantly, the persistence of *intI1* across the broader time series aligns with its established role as a conservative marker of anthropogenic impact and genetic mobility in wastewater environments, where it often exhibits lower removal efficiency and greater stability than many ARG targets [8, 47].

The *ermB* gene showed plant-specific fluctuations during the initial monitoring period, with abundances ranging from 1.4 to 2.5 log copies mL⁻¹. EPC exhibited numerically higher early concentrations, whereas CJC showed a pronounced transient decrease in January 2022 (~ 0.1 log copies mL⁻¹), with values lower than those recorded in December 2021 and February 2022 (both adjusted *p* < 0.001). Between early 2022 and August 2022, concentrations increased from January to August at EPC (adjusted *p* = 0.0197), remained similar from February to August at CJC (adjusted *p* = 1.000), and increased from February to August at JW (adjusted *p* < 0.001). From October 2022 onward, the observed concentration ranges became more similar among the three WWTPs (~ 1.4–1.7 log copies mL⁻¹), although within-plant fluctuations persisted (Fig. 1c). During 2023, JW showed a transient increase in March, with concentrations higher than those recorded in February and April 2023 (both adjusted *p* < 0.001).

Similar temporal fluctuations in *ermB* abundance have been reported in full-scale WWTPs and associated with wastewater composition, bacterial community dynamics, and particulate association [36, 48]. Some of the higher *ermB* concentrations in the present study occurred at selected sampling points during the COVID-19 pandemic, when macrolides, particularly azithromycin, were widely used in clinical practice for COVID-19 management [49]. However, because local antimicrobial prescription, consumption, and wastewater antimicrobial-residue data were unavailable, this temporal overlap cannot be interpreted as evidence of population-level macrolide-driven selective pressure. Moreover, *ermB* showed plant-specific fluctuations after September 2022 rather than a uniform gradual decline across the three WWTPs. Thus, the observed patterns do not demonstrate progressive relaxation of antimicrobial selective pressure and may reflect the combined effects of bacterial abundance, wastewater composition, community dynamics, hydrological conditions, and other unmeasured factors [44, 50].

Normalization of *intI1* and *ermB* by the 16 S rRNA gene helped contextualize whether changes in absolute marker concentrations occurred in parallel with variations in the overall bacterial signal or in their relative abundances within the microbial community. Across wastewater studies, relative abundances normalized to 16 S are widely used to distinguish changes in total bacterial abundance from changes in the relative representation of resistance markers [44, 46, 50, 51].

**Fig. 2:**
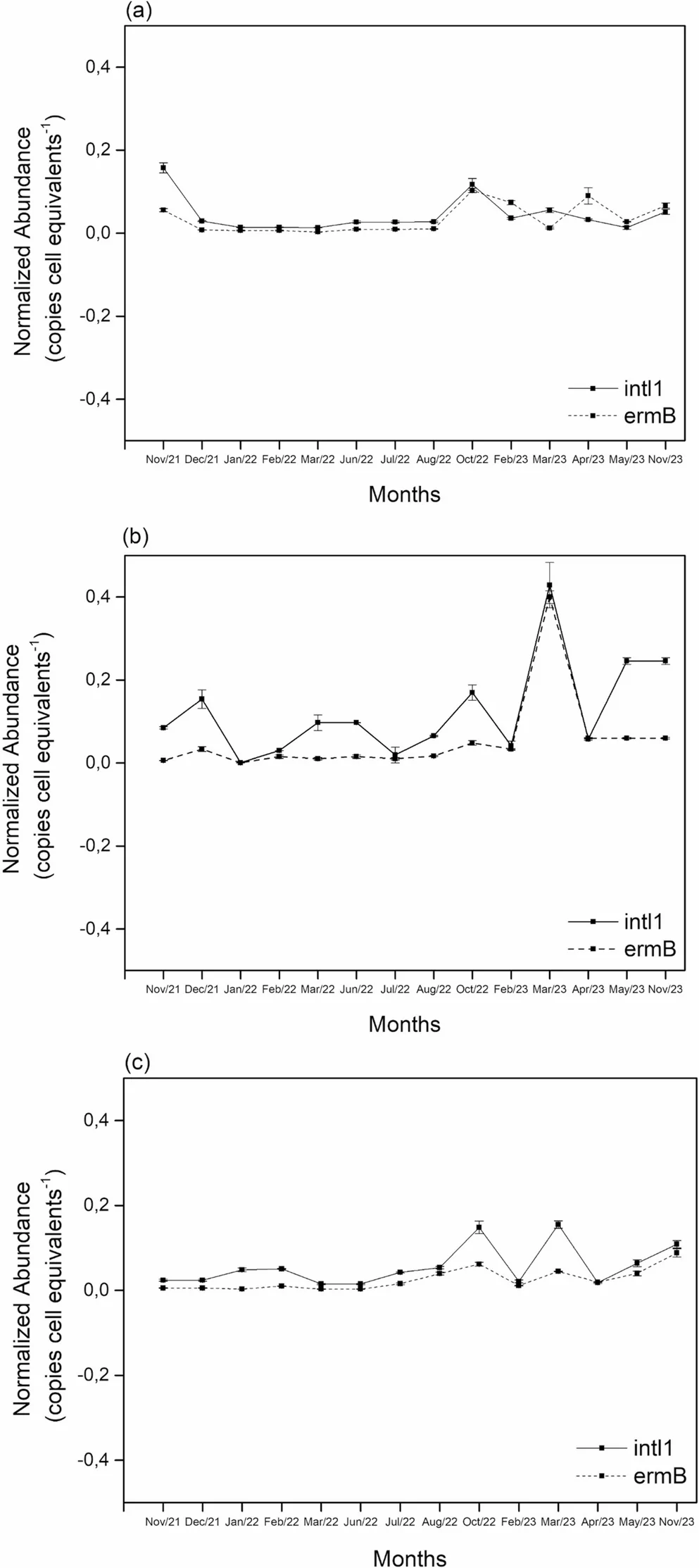
Normalized abundance (copies cell equivalents^− 1^) of *intI1* and *ermB* genes determined by qPCR in wastewater samples from the EPC (**a**), CJC (**b**), and JW (**c**) treatment plants between November 2021 and November 2023. Gene copy numbers were normalized to 16S rRNA gene abundance to account for variations in total bacterial load. Data points represent mean values of technical replicates and error bars indicate standard deviation

At EPC, both normalized markers remained generally low, mostly within approximately 0.0–0.1 copies per cell equivalent, throughout the monitoring period (Fig. 2a). Although isolated increases were observed, particularly for *intI1* in November 2021 and October 2022, the two markers showed comparatively limited normalized variation during most of the time series. The near-parallel trajectories observed at several sampling points suggest that changes in absolute marker concentrations frequently occurred alongside changes in the overall bacterial signal. However, the normalized proportions should not be considered constant throughout the entire monitoring period.

In contrast, CJC exhibited the broadest observed range of normalized abundances. Both *intI1* and *ermB* reached approximately 0.4 copies per cell equivalent in March 2023 (Fig. 2b). After this shared peak, the markers followed different trajectories: *intI1* subsequently increased to approximately 0.25 in May and November 2023, whereas *ermB* returned to approximately 0.05. The co-occurrence of the March 2023 increases indicates a transient elevation in the marker-to-16 S ratios within the analyzed sample, although the underlying environmental or biological driver could not be determined. The magnitude of these normalized values falls within the range reported for full-scale effluents in other longitudinal studies [36, 48].

JW displayed an intermediate pattern, with normalized abundances generally ranging from approximately 0.0 to 0.16 copies per cell equivalent (Fig. 2c). Moderate temporal oscillations were observed, particularly for *intI1*, with increases in October 2022 and March 2023, but without the pronounced simultaneous peak in both markers observed at CJC. This observed gradation—EPC generally low, JW intermediate, and CJC showing the largest peaks—describes the normalized profiles of the analyzed samples but should not be interpreted as a statistically demonstrated difference in stability or variability among WWTPs.

Collectively, the dataset supports three principal inferences. First, the absolute qPCR results showed a decline in 16 S rRNA gene concentrations across all three WWTPs between August and October 2022, whereas *intI1* showed decreases at CJC and JW but not at EPC. Second, normalization showed that the relative abundances of the markers were generally low at EPC, intermediate at JW, and characterized by larger isolated peaks at CJC. These normalized profiles indicate that some changes in absolute marker concentrations occurred alongside changes in the marker-to-16 S ratios, whereas others may have reflected variation in the overall bacterial signal. Third, the continued detection of *intI1* throughout the time series reinforces its suitability as an indicator of anthropogenic influence and class 1 integron occurrence in full-scale wastewater systems [47]. A fourth inference emerges when the time series is interpreted within the pandemic–post-pandemic framework. Some higher and more variable absolute *ermB* abundances were observed at selected sampling points during the pandemic, followed by plant-specific trajectories and more similar concentration ranges among the WWTPs after September 2022. This temporal pattern is consistent with reports of increased macrolide use during the COVID-19 pandemic [49]. However, because local antimicrobial-consumption data were not available, this association should be regarded as a plausible hypothesis rather than direct evidence of changes in antibiotic selective pressure. These findings nevertheless illustrate the potential of wastewater surveillance to track temporal variation in targeted AMR markers within a One Health framework [45, 50].

Overall, the longitudinal analysis revealed distinct temporal trajectories of 16 S rRNA, *intI1*, and *ermB* across the three WWTPs. Absolute 16 S rRNA gene concentrations declined across all systems in late 2022, whereas *intI1* and *ermB* showed plant-specific fluctuations. Normalization further demonstrated that part of the observed variability was accompanied by changes in the relative abundance of the markers, whereas other changes occurred in parallel with the overall bacterial signal. Together, these findings establish the temporal framework for interpreting subsequent differences in microbial community structure and their relationship with the target genes across the studied wastewater systems. They also reinforce the value of longitudinal wastewater surveillance for tracking temporal changes in targeted environmental AMR markers, while recognizing that the underlying population-level or environmental drivers were not directly evaluated [44, 52].

### Physicochemical context

**Fig. 3:**
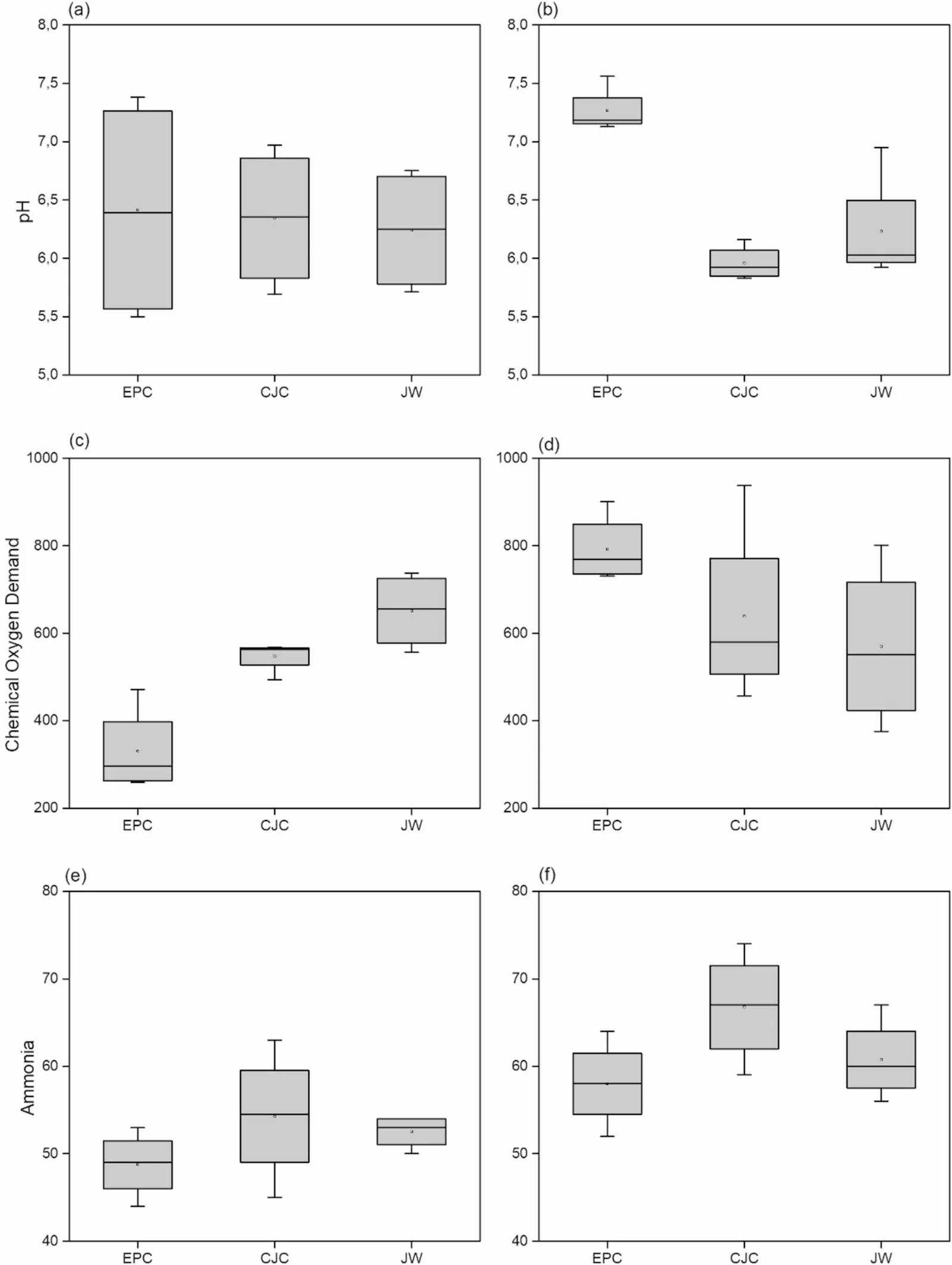
Distribution of physicochemical parameters in wastewater from the EPC, CJC, and JW treatment plants during the monitoring period. Boxplots represent pH (**a**–**b**), chemical oxygen demand (COD) (**c**–**d**), and ammonia concentrations (**e**–**f**) measured under two sampling phases. Boxes indicate the interquartile range, horizontal lines represent medians, whiskers denote minimum–maximum values, and dots indicate mean values

To provide environmental context for the molecular analyses, physicochemical parameters were evaluated throughout the study. The three wastewater treatment plants exhibited substantial spatial heterogeneity, particularly during the early phase of the monitoring period. In December 2021, EPC displayed a broader pH interval (≈ 5.5–7.3) compared with the narrower ranges observed at CJC and JW (≈ 6.1–6.9), suggesting greater short-term variability in influent composition at EPC. This separation became more evident in January 2022, when EPC maintained consistently higher pH values (≈ 7.0–7.2) while CJC and JW shifted toward more acidic conditions (Fig. 3a, b). Differences of this magnitude among plants are consistent with reports showing that wastewater pH can reflect variations in source contributions, dilution, and biochemical activity within sewer catchments rather than plant operational conditions [53, 54]. Within wastewater monitoring frameworks, pH is frequently interpreted as an indicator of changing organic inputs and microbial metabolism, reinforcing that inter-station contrasts can arise from upstream ecological dynamics.

Chemical oxygen demand (COD) exhibited even clearer stratification in December 2021, with a gradient from EPC (≈ 250–400 mg L⁻¹) to CJC (≈ 550–600 mg L⁻¹) and JW (≈ 650–700 mg L⁻¹) (Fig. 3c, d). Such separation indicates distinct organic loading regimes among the catchments and aligns with studies demonstrating that COD functions as a sensitive proxy for population-derived inputs and metabolic activity in wastewater [55, 56]. In January 2022, COD increased across all stations and the ranges overlapped substantially, eliminating statistical separation. The convergence suggests a temporary homogenization of organic loads, potentially driven by shared seasonal or hydrological influences. Similar temporal compression of inter-plant differences has been documented in longitudinal wastewater surveys, where external drivers such as rainfall or regional behavioral shifts synchronize influent composition across systems [57].

Ammonia concentrations did not differ statistically among stations within each month, yet their temporal behavior revealed important ecological signals. December 2021 values were consistently lower than those measured in January 2022, with the most pronounced increase occurring at CJC (Fig. 3e, f). Ammonium has been repeatedly shown to act as a stable marker of human-derived nitrogen inputs and population-equivalent loading in wastewater systems [56, 58]. The increase observed in January 2022 is therefore compatible with a higher nitrogen load during this sampling period, although the available data do not distinguish changes in catchment inputs from other factors such as dilution, wastewater residence time, or temporal variability in influent composition [53, 57].

Taken together, the abiotic data indicate that EPC had higher pH in January 2022, while COD followed a plant-specific gradient in December 2021, with the highest values at JW, followed by CJC and EPC. Ammonia increased temporally, particularly at CJC, but did not differ significantly among WWTPs within either month. Thus, the physicochemical profiles demonstrate both plant-specific and temporal variation rather than a fixed classification of EPC, CJC, and JW into consistently distinct environmental regimes. This spatial structuring mirrors findings from long-term regional monitoring in the city of Fortaleza, where physicochemical variability among EPC, CJC, and JW was attributed to differences in catchment vulnerability, urban density, and inflow dynamics [54].

The exploratory analysis did not identify statistically significant correlations between the physicochemical parameters and the normalized abundances of the antimicrobial resistance markers, although the estimated coefficients varied in magnitude and direction.

For *ermB*, pH showed a moderate positive Spearman coefficient (ρ = 0.60; *p* = 0.35), whereas COD showed a moderate negative coefficient (ρ = −0.60; *p* = 0.35). Ammonia exhibited the strongest coefficient, with a negative association with normalized *ermB* abundance (ρ = −0.90; *p* = 0.083). Because none of these correlations reached statistical significance and only six paired observations were available, their direction and magnitude should be interpreted as exploratory patterns rather than evidence of physicochemical control of *ermB* abundance. Simple linear regression models showed comparable patterns. The model using pH as an explanatory variable had limited explanatory power (R² = 0.18; *p* = 0.475), while COD showed a weak negative relationship with normalized *ermB* abundance (R² = 0.06; *p* = 0.686). The ammonia-based model explained a larger proportion of the observed variance (R² = 0.64), although the relationship was not statistically significant (*p* = 0.102). This result identifies ammonia as a variable of potential interest for subsequent investigation but does not demonstrate a statistically supported relationship in the present dataset.

For *intI1*, correlations with physicochemical parameters were also not statistically significant. pH showed a weak positive correlation (Spearman ρ = 0.20; *p* = 0.95), whereas COD exhibited a moderate negative correlation (ρ = −0.60; *p* = 0.35). Ammonia concentration showed a moderate negative correlation with *intI1* abundance (ρ = −0.50; *p* = 0.45). Simple linear regression models similarly indicated no significant associations between physicochemical parameters and *intI1* abundance. The pH model showed limited explanatory power (*R*² = 0.055; *p* = 0.703), as did the COD model (*R*² = 0.105; *p* = 0.595). Although the ammonia model showed a negative trend, it was not statistically significant (*R*² = 0.49; *p* = 0.191).

Overall, the physicochemical parameters evaluated in the paired dataset were not statistically associated with the normalized relative abundances of *ermB* or *intI1*. The negative coefficients observed for ammonia, particularly in relation to *ermB*, should therefore be treated as hypothesis-generating patterns that require evaluation using larger datasets and additional sampling periods.

Together, these physicochemical patterns provide environmental context for interpreting the temporal variation observed in the molecular and microbial data. pH, COD, and ammonia reflect spatial and temporal differences in wastewater composition and may represent potential environmental covariates in wastewater surveillance. The present study directly evaluated their relationships with the normalized marker abundances, but the small, paired dataset and absence of statistically significant associations limit the conclusions that can be drawn. Accordingly, the physicochemical measurements provide contextual information for the microbiological analyses rather than evidence that the evaluated variables drove variation in *ermB*, *intI1*, or bacterial community structure [53, 55].

### Bacterial community structure

A total of 1,573,002 raw reads were generated. Following quality filtering, denoising, paired-end merging, and chimera removal, 1,173,633 high-quality non-chimeric reads were retained, corresponding to an overall retention rate of 74.61%. Final sequencing depth ranged from 40,512 to 104,562 reads per sample, with a mean of 65,202 reads per sample. No samples were excluded based on sequencing depth.

The three WWTP wastewater communities share a conserved taxonomic backbone dominated by the bacterial phyla Bacillota, Pseudomonadota, and Bacteroidota (Fig. 4a), a structure repeatedly reported as a core signature of municipal wastewater microbiomes worldwide [59, 60]. Similar phylum-level dominance has been documented in Brazilian WWTPs operating under different technological configurations, reinforcing that wastewater communities retain a stable fecal-derived microbial fingerprint despite process variation [36]. These phyla are strongly associated with organic matter degradation and human gut microbiota, reflecting both influent origin and ecological filtering occurring within wastewater environments [59, 60]. Although a shared phyla core was evident, clear plant-specific structuring occurred. EPC displayed broader phylogenetic diversity and relative enrichment of Campylobacterota, whereas JW and CJC showed more compressed community profiles (Fig. 4a). Such cross-plant heterogeneity is consistent with evidence that catchment size, population served, and system-scale operational conditions shape microbial community assembly in WWTP effluents [36, 44, 61]. However, the taxonomic profiles alone do not identify which of these factors caused the differences observed among EPC, CJC, and JW.Fig. 4Microbial community structure of wastewater samples from the EPC, CJC, and JW wastewater treatment plants based on 16 S rRNA gene sequencing during the two sequenced sampling periods (December 2021 and January 2022). (**a**) Relative abundance of the dominant bacterial phyla across wastewater treatment plants and sampling periods. (**b**) Heatmap showing genus-level relative abundance patterns among samples. (**c**) Observed ASV richness, (**d**) Chao1 estimated richness, (**e**) Shannon diversity index, and (**f**) inverse Simpson diversity index. Statistical differences in alpha-diversity metrics among wastewater treatment plants were assessed using one-way ANOVA or Kruskal–Wallis tests, with pairwise comparisons indicated by significance levels. (**g**) Principal coordinates analysis (PCoA) based on weighted UniFrac distances illustrating beta-diversity relationships among wastewater microbial communities

**Figure:**
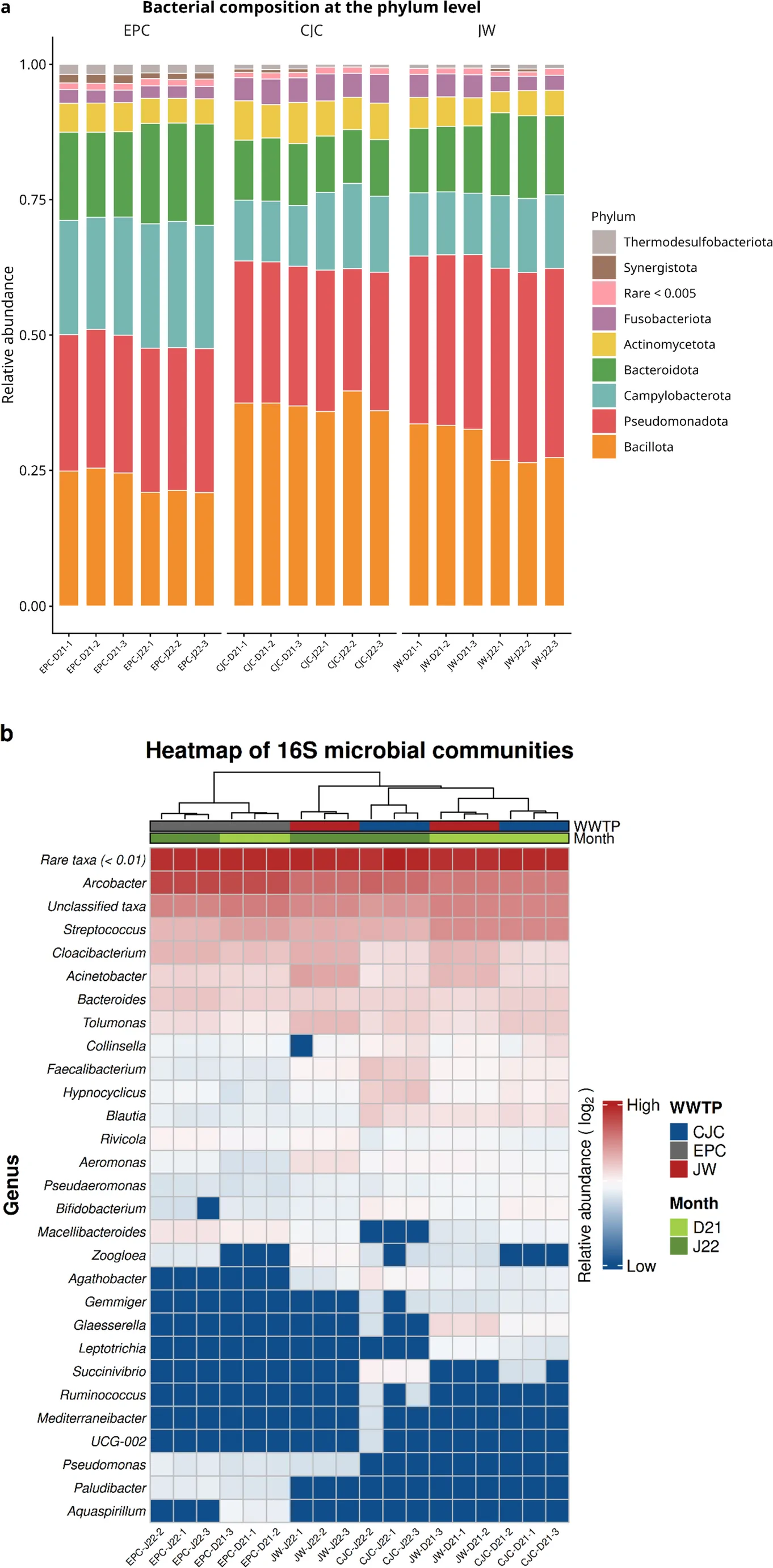

**Figure:**
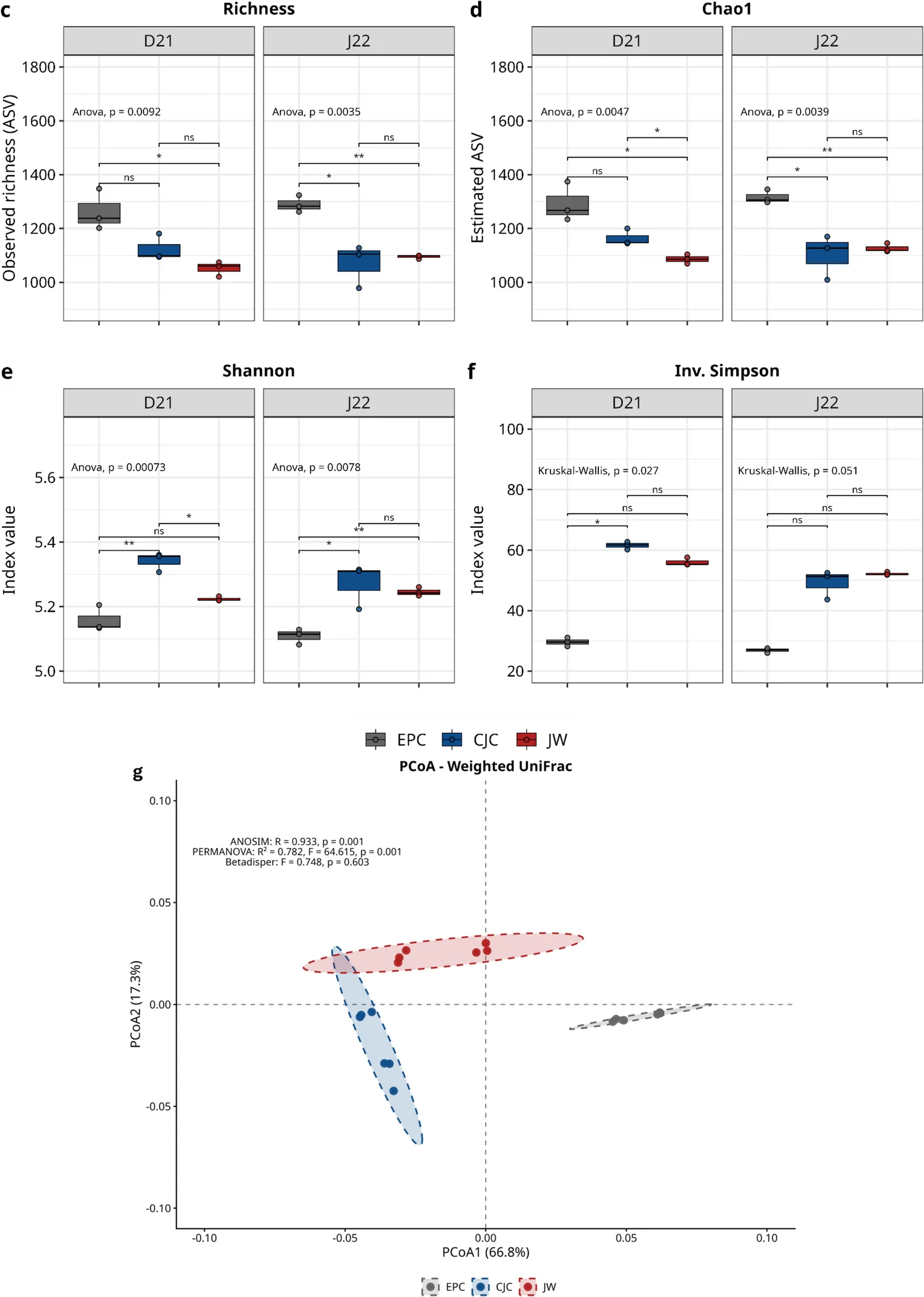

At the genus level, the treated effluents exhibited a persistent human-associated microbial signature, characterized by the consistent detection of *Arcobacter*, *Streptococcus*, *Cloacibacterium*, *Acinetobacter*, *Bacteroides*, and *Tolumonas*. (Fig. 4b), mirroring previous wastewater surveys in which gut-derived taxa persist throughout wastewater processing [36, 60, 62–64]. Hierarchical clustering further revealed that samples grouped predominantly according to WWTP rather than sampling period, indicating that plant-specific operational and environmental conditions exerted a stronger influence on bacterial community composition than temporal variation. Although these core wastewater-associated genera were consistently detected across all WWTPs, several taxa displayed plant-specific enrichment patterns, contributing to the distinct microbial signatures of each treatment plant. Among the classified genera, *Arcobacter* emerged as the dominant taxon, particularly in EPC (Fig. 4b), a pattern frequently reported in wastewater systems where this genus is recognized as an indicator of fecal contamination and environmental persistence [36, 59, 65]. Its predominance, together with the persistence of low-abundance taxa across all samples, suggests that treated effluents harbor both a stable core microbiome and a reservoir of microbial diversity, reflecting the ecological resilience of wastewater bacterial communities despite differences in treatment plants and sampling periods [66].

A substantial fraction of sequences remained taxonomically unresolved across the wastewater systems and sampling periods (Fig. 4b). This pattern is widely recognized in wastewater microbiome studies and reflects the incomplete representation of WWTP-adapted lineages in universal reference databases rather than biological randomness. Ecosystem-specific frameworks such as the MiDAS (Microbial Database for Activated Sludge) catalogue demonstrate that WWTPs host thousands of recurrent but previously unnamed taxa that require dedicated taxonomic scaffolding for proper resolution [67]. Brazilian datasets also report substantial proportions of unclassified sequences when short-read amplicons are used, reinforcing that database coverage remains a limiting factor in environmental annotation [36]. Full-length 16 S rRNA approaches substantially increase classification rates and reveal stable WWTP-specific clades hidden in short-read surveys [59].

Alpha-diversity metrics showed distinct patterns among WWTPs, revealing contrasting trends in richness and community evenness across sampling periods (Fig. 4c–f). Overall differences among WWTPs were significant for observed richness (Observed ASVs) in D21 (ANOVA, *p* = 0.0092) and J22 (ANOVA, *p* = 0.0035), as well as for estimated richness (Chao1) in D21 (ANOVA, *p* = 0.0047) and J22 (ANOVA, *p* = 0.0039). EPC consistently exhibited the highest observed and estimated richness, whereas CJC showed intermediate richness values and JW generally displayed the lowest richness (Fig. 4c, d). In contrast, Shannon diversity was significantly higher in CJC than in EPC during both sampling periods (Fig. 4e), and inverse Simpson values followed the same trend, although differences were significant only in D21 (Fig. 4f). These findings indicate that, despite harboring a greater number of taxa, EPC was characterized by lower community evenness, reflecting the marked dominance of *Arcobacter*. Conversely, the higher Shannon and inverse Simpson values observed in CJC indicate a more equitable distribution of taxa, resulting in greater overall community diversity despite its lower richness.

Beta diversity was evaluated using Weighted UniFrac, Unweighted UniFrac, and Bray–Curtis distances. Weighted UniFrac ordination revealed clear separation of bacterial communities according to WWTP, with the first two principal coordinate axes explaining 66.8% and 17.3% of the total variation, respectively (Fig. 4g). EPC formed a more compact cluster, whereas CJC and JW exhibited broader visual distributions. However, because multivariate dispersion did not differ significantly among WWTPs (Betadisper, *F* = 0.748, *p* = 0.603), these differences in cluster width should not be interpreted as differences in within-group variability. PERMANOVA confirmed that WWTP was the primary factor structuring bacterial community composition, explaining 78.2% of the variation (*R*² = 0.782, *F* = 64.615, *p* = 0.001), while sampling period also had a significant but smaller effect, accounting for 13.3% of the variation (*R*² = 0.133, *F* = 21.989, *p* = 0.001). Consistent with these results, ANOSIM indicated strong separation among WWTPs (*R* = 0.933, *p* = 0.001).

Unweighted UniFrac, which considers only taxon presence and absence, also identified WWTP as a significant determinant of bacterial community composition, explaining 25.3% of the variation (*R*² = 0.253; *F* = 2.629; *p* = 0.001). In contrast, sampling period was not statistically significant, although it approached the significance threshold (*R*² = 0.075; *F* = 1.558; *p* = 0.057). Bray–Curtis distances revealed a similar pattern, with WWTP explaining 63.5% of the variation in community composition (*R*² = 0.635; *F* = 17.603; *p* = 0.001), whereas sampling period accounted for a smaller but significant proportion of the variation (*R*² = 0.113; *F* = 6.273; *p* = 0.004). Collectively, these complementary analyses consistently identified WWTP as the primary driver of bacterial community structure. The influence of sampling period was comparatively weaker and depended on the distance metric, being significant when community abundance was considered (Weighted UniFrac and Bray–Curtis), but not when analyses were based solely on taxon presence or absence (Unweighted UniFrac).

Multivariate dispersion analyses revealed no significant differences in within-group variability for any of the distance metrics evaluated, including Bray–Curtis (ANOVA: *F* = 0.372, *p* = 0.858), Unweighted UniFrac (ANOVA: *F* = 2.151, *p* = 0.129), and Weighted UniFrac (ANOVA: *F* = 0.748, *p* = 0.603) (Fig. 4g). These results indicate that the significant PERMANOVA findings reflect genuine differences in bacterial community composition and phylogenetic structure among WWTPs rather than artifacts arising from unequal multivariate dispersion. Similar WWTP-specific patterns of beta diversity have been reported in longitudinal wastewater studies, where differences in microbial community structure have been linked to variations in catchment characteristics, influent composition, treatment processes, and operational conditions [36, 44, 61, 68, 69]. 

In the integrated analysis restricted to samples with paired microbiome, qPCR, and physicochemical data, bacterial community composition was primarily associated with WWTP (R² = 0.692; *p* = 0.001), whereas sampling period had no significant effect (R² = 0.119; *p* = 0.158). When evaluated individually, neither *ermB* nor *intI1* abundance was significantly associated with bacterial community structure (*ermB*: R² = 0.141, *p* = 0.097; *intI1*: R² = 0.101, *p* = 0.211). However, after accounting for the effect of WWTP in a marginal model, *intI1* explained a significant additional proportion of the variation in bacterial community composition (R² = 0.096; *F* = 5.012; *p* = 0.022), indicating that differences in *intI1* abundance were associated with community structure independently of plant identity. When both resistance markers were included simultaneously with WWTP, neither *ermB* nor *intI1* showed a significant independent association with bacterial community composition (*ermB*: R² = 0.027, *p* = 0.232; *intI1*: R² = 0.029, *p* = 0.210), whereas WWTP remained the dominant explanatory factor (R² = 0.515; *F* = 13.932; *p* = 0.001). Overall, these findings do not support a consistent relationship between the abundance of the two resistance markers and bacterial community structure. Rather, they suggest that the association between *intI1* and community composition is weak and context-dependent, becoming significant only after adjustment for WWTP in a single model and disappearing when both markers were evaluated simultaneously.

These ecological patterns are relevant to wastewater-based AMR surveillance because they demonstrate that bacterial community composition differs substantially among WWTPs. Previous studies have reported associations between bacterial community structure, antimicrobial resistance genes (ARGs), and mobile genetic elements in full-scale wastewater treatment plants [44, 70]. Gut-associated and potentially opportunistic genera, including *Arcobacter* and *Acinetobacter*, were consistently detected throughout the study. Although these genera have been reported in wastewater environments where ARGs and class 1 integrons occur [36], in the present study, their co-occurrence with *ermB* and *intI1* does not demonstrate that these genera carried either marker or contributed directly to their persistence, as 16 S rRNA amplicon sequencing provides taxonomic, but not functional or strain-level, resolution. Establishing such associations would require approaches such as shotgun metagenomics, genome-resolved analyses, or culture-based characterization of isolates.

Brazilian and international studies have shown that ARGs and class 1 integrons can persist throughout wastewater treatment processes and remain detectable in treated effluents [36, 62, 71]. Our findings support the value of integrating bacterial community profiling with targeted qPCR markers to provide ecological context for AMR surveillance. However, this approach does not allow inference of functional resistance, horizontal gene transfer, or specific associations between ARGs and their bacterial hosts.

### Integrated analysis of bacterial community composition and AMR markers

Previous longitudinal studies of full-scale WWTPs have reported associations between bacterial community structure, antimicrobial resistance genes (ARGs), and mobile genetic elements [36, 44, 61]. These findings support the interpretation of targeted AMR markers within the broader ecological context of the wastewater microbiome rather than solely as reflections of total bacterial abundance [36, 70]. In the present study, however, the integrated analyses showed that bacterial community composition was influenced primarily by WWTP identity, whereas the associations between community structure and the targeted markers (*ermB* and *intI1*) were weak and not consistently supported across statistical models.

Among the molecular markers evaluated, *intI1* deserves particular attention because it is widely recognized as an indicator of anthropogenic impact and the occurrence of class 1 integron-associated elements in wastewater environments. Previous studies have reported the persistence of *intI1* in wastewater communities containing opportunistic and environmentally persistent taxa, including *Arcobacter*, *Acinetobacter*, and members of the Enterobacteriaceae [36, 70]. In the present study, however, neither *ermB* nor *intI1* showed a consistent association with bacterial community composition. Although *intI1* explained a small but significant proportion of community variation after adjustment for WWTP in one marginal model, this effect was not retained when both resistance markers were included simultaneously. These findings indicate that the association between *intI1* and bacterial community structure was weak and dependent on the statistical model, rather than representing a robust ecological relationship.

Several gut-associated or potentially opportunistic genera, including *Arcobacter*, *Acinetobacter*, *Bacteroides*, *Prevotella*, and *Streptococcus*, were detected in the analyzed wastewater communities. *Arcobacter* increased in relative abundance between D21 and J22 across all three WWTPs, whereas *Acinetobacter* exhibited plant-specific variation. Previous studies have reported the occurrence of *Arcobacter* and *Acinetobacter* in wastewater environments where ARGs and class 1 integrons are prevalent [36], while *Bacteroides*, *Prevotella*, and *Streptococcus* are recognized as major contributors to the fecal microbial signature of municipal wastewater [62, 65]. Although community-level associations were evaluated, this study did not assess direct relationships between individual bacterial genera and *ermB* or *intI1*, nor did it identify the bacterial hosts of these markers. Therefore, the co-occurrence of bacterial taxa and molecular markers within the same wastewater samples should not be interpreted as evidence that the detected genera carried these markers or contributed directly to their persistence. Previous network analyses of Brazilian WWTPs have identified associations among bacterial community composition, ARG abundance, and class 1 integrons, supporting the value of integrating microbiome profiling with targeted molecular surveillance [36]. However, these observations provide ecological context and should not be extrapolated to infer equivalent taxon–marker associations in the present dataset.

Overall, the integrated analyses showed that bacterial community composition was primarily structured by differences among WWTPs, whereas the targeted resistance markers exhibited weak and context-dependent associations with the microbiome. The influence of sampling period was comparatively smaller and varied according to the analytical dataset and distance metric, being significant in the overall beta-diversity analyses but not in the paired integrated analysis. Accordingly, the data do not support a consistent temporal coupling between changes in *ermB* and *intI1* abundance and shifts in bacterial community composition. Instead, microbiome profiling provides ecological context for interpreting targeted AMR markers while indicating that WWTP-specific characteristics and other unmeasured environmental or operational factors are likely to account for much of the observed variation.

From a One Health perspective, these findings highlight the value of integrating bacterial community profiling with targeted molecular surveillance to improve the ecological interpretation of AMR markers in wastewater. Although this approach does not establish functional resistance, horizontal gene transfer, or ARG–host associations, it provides a robust framework for interpreting targeted AMR markers within complex microbial communities. Future studies integrating longitudinal molecular surveillance, shotgun metagenomics, environmental variables, and broader spatial and temporal sampling will further improve our understanding of AMR persistence and dissemination in wastewater systems [36, 71].

## Conclusions

By integrating longitudinal microbiome profiling with quantitative measurements of *ermB* and *intI1* across three full-scale WWTPs during and after the COVID-19 pandemic, this study provides new insights into how wastewater treatment plants influence the persistence and temporal dynamics of targeted AMR markers under real-world operational conditions. Although the data do not support a direct coupling between bacterial community composition and the temporal dynamics of these markers, they demonstrate that WWTP identity is the primary factor structuring bacterial communities, whereas the persistence of *ermB* and *intI1* is likely influenced by a combination of environmental and operational factors rather than by community composition alone. Consequently, the present findings suggest that WWTPs function as dynamic microbial ecosystems in which the persistence of targeted AMR markers reflects complex ecological processes rather than acting simply as reservoirs or ecological filters.

Overall, these findings highlight the value of integrating targeted molecular markers with bacterial community profiling for wastewater-based AMR surveillance while recognizing the limitations of targeted gene analyses for inferring functional resistance or ARG–host associations. Future implementation of wastewater surveillance in Brazil could benefit from a sentinel network of WWTPs integrated, where feasible, with longitudinal environmental measurements and complementary epidemiological information, including antimicrobial prescription or consumption records. Such integration could strengthen national One Health strategies for long-term surveillance of environmental AMR markers.

## Data Availability

Data supporting the findings of this study are mostly presented in the article. Any further relevant data are available from the corresponding author upon reasonable request.
